# Novel, Moon and Mars, partial gravity simulation paradigms and their effects on the balance between cell growth and cell proliferation during early plant development

**DOI:** 10.1038/s41526-018-0041-4

**Published:** 2018-04-04

**Authors:** Aránzazu Manzano, Raúl Herranz, Leonardus A. den Toom, Sjoerd te Slaa, Guus Borst, Martijn Visser, F. Javier Medina, Jack J. W. A. van Loon

**Affiliations:** 1 Centro de Investigaciones Biológicas (CSIC), Madrid, Spain; 2 DESC (Dutch Experiment Support Center), Department of Oral and Maxillofacial Surgery / Oral Pathology, VU University Medical Center & Academic Centre for Dentistry Amsterdam (ACTA), Amsterdam Movement Sciences, Amsterdam, The Netherlands; 3 European Space Agency Technology Center (ESA-ESTEC), TEC-MMG LIS Lab, Noordwijk, The Netherlands; 4 Department of Medical Technology, VU University Medical Center, Amsterdam Movement Sciences, Amsterdam, The Netherlands; 5 Airbus Defense and Space, Leiden, The Netherlands

## Abstract

Clinostats and Random Positioning Machine (RPM) are used to simulate microgravity, but, for space exploration, we need to know the response of living systems to fractional levels of gravity (partial gravity) as they exist on Moon and Mars. We have developed and compared two different paradigms to simulate partial gravity using the RPM, one by implementing a centrifuge on the RPM (RPM^HW^), the other by applying specific software protocols to driving the RPM motors (RPM^SW^). The effects of the simulated partial gravity were tested in plant root meristematic cells, a system with known response to real and simulated microgravity. Seeds of *Arabidopsis thaliana* were germinated under simulated Moon (0.17 *g*) and Mars (0.38 *g*) gravity. In parallel, seeds germinated under simulated microgravity (RPM), or at 1 *g* control conditions. Fixed root meristematic cells from 4-day grown seedlings were analyzed for cell proliferation rate and rate of ribosome biogenesis using morphometrical methods and molecular markers of the regulation of cell cycle and nucleolar activity. Cell proliferation appeared increased and cell growth was depleted under Moon gravity, compared with the 1 *g* control. The effects were even higher at the Moon level than at simulated microgravity, indicating that meristematic competence (balance between cell growth and proliferation) is also affected at this gravity level. However, the results at the simulated Mars level were close to the 1 *g* static control. This suggests that the threshold for sensing and responding to gravity alteration in the root would be at a level intermediate between Moon and Mars gravity. Both partial *g* simulation strategies seem valid and show similar results at Moon *g*-levels, but further research is needed, in spaceflight and simulation facilities, especially around and beyond Mars g levels to better understand more precisely the differences and constrains in the use of these facilities for the space biology community.

## Plant biology: partial gravity impacts root development

Novel simulators of partial gravity show that the threshold for plants to sense and respond to gravity in their roots lies somewhere between gravity levels of the Moon and Mars. An international team led by Jack van Loon from the VU University Medical Center in Amsterdam, the Netherlands, reconfigured the hardware (include centrifugation) and software (new algorithm) on a microgravity-simulating instrument known as a Random Positioning Machine to produce fractional levels of gravity similar to those found on Mars (0.38 *g*) and the Moon (0.17 *g*). They germinated seeds of thale cress (*Arabidopsis thaliana*) in these devices, and showed in 4-day old seedlings that the balance between cell proliferation and cell growth in the roots was out of whack in both simulated microgravity and Moon gravity levels, but not in a simulated Mars scenario.

## Introduction

Since the beginning of life on Earth, organisms have evolved in an environment of physico-chemical factors, many of them changing (pressure, temperature, humidity,…) while a few remained constant or almost constant, such as the gravitational and magnetic fields.^1^ Therefore, all living organisms are well adapted to the different conditions on Earth.

However, despite the constancy in direction and magnitude of the gravity vector on Earth, it is well known that plants respond to gravity alterations. Gravity influences the direction of plant growth and the pattern of development, from seedlings to adult plants,^2^ and even gravitational effects on in vitro plant cell cultures have been reported.^3,4^ Studies on the response of living beings to altered gravity are greatly facilitated by the development of ground-based facilities for simulation of gravity alterations to perform basic science as well as to design and prepare for space experiments.

Tampering with a force like gravity can only be done manipulating its magnitude or its direction because one cannot cancel the force of gravity, for long periods, while remaining on the surface of a celestial body like Earth. Decreasing its magnitude can be done by moving into a field with a lower acceleration like Moon or Mars partial gravity, or fall around a celestial body with the same acceleration as generated by that of the celestial body, a situation know as free fall, also referred to as microgravity or (better) near weightlessness. For providing free fall on or from Earth this means making use of, e.g., a parabolic flight (PF) or a drop tower. The main disadvantage of the latter two facilities is the limited time for such a fall. For aircraft PF this is in the order of ten to some 50 s depending on the aircraft and this time increases up to some 13 min by using ballistic rockets like the MAXUS system.^5^ Note that for the latter two conditions also periods of hypergravity are involved which might distort the actual microgravity effect.^6^ An alternative is to compensate gravity by levitating individual atoms by means of a strong gradient magnetic field.^7,8^ However, one has to take into account the strong magnetic field as such, this is particularly relevant for the case of biological experiments.^9,10^

Over the years, especially plant researchers directed their efforts to simulate near weightlessness more and more toward manipulating the direction of the gravity vector, as was initiated by von Sachs who also introduced the term ‘klinostat’ for such devices.^11^ Various forms of clinostats were developed where one or more samples were rotated around their longitudinal horizontal axis. The simulation of microgravity with clinostats allowed the start of development of partial gravity paradigms. Partial, fractional or hypo-gravity is referred to for any *g* level between the theoretical zero up to Earth’s unit gravity of 9.81 ms^−2^ (1 *g*).

The development of such partial-*g* clinostats was initially driven by the interest to determine the gravity threshold of plants and fungi. What is the minimum gravity stimulus required to trigger a tropic response in plants? One of the earlier and very elegant 2-axis clinostat systems built for threshold studies was described by Shen-Miller and colleagues^12^ but also a relatively large diameter centrifuge outfitted with clinostats was used to address the threshold issue.^13^

Other researchers applied a simple angled or inclined clinostat to generate partial gravity (see, e.g.^14,15^) and also systems like the centrifuge-clinostats were constructed, as shown by Laurinavicius et al.^16^ or Galland et al.^17^ All these systems have in common that the microgravity or near weightlessness simulation was performed by rotating the sample around its longitudinal axis. However, such one-axis clinostats have their limitations, especially in plant related studies, as has been pointed out by, e.g., Brown et al.^15^. Some years ago, we also demonstrated that, focusing on plastid position, a 3D simulation seems to mimic real near weightlessness more closely than a 2D simulation.^18^ Researchers started to develop 3D or two-axes simulation systems where one of the very first systems of such kind was described by Rudolf Magnus^19^ for vestibular studies in rabbits in the beginning of the 20th century. Later similar systems were introduced in plants studies see, for overview,^20^. From regular steady rotation as commonly used for clinostat systems one introduced random motions as simulation which resulted in the so called random positioning machines (RPM).^21,22^

In recent years more interest has developed in partial gravity research. In addition to the intrinsic value of these studies in *g*-threshold responses, more emphasis has been put on the connection of these investigations with the various future space exploration programs with an interest to put plants and humans on Moon and Mars.^23,24^ It is worth mentioning that partial gravity studies using plants have been carried out recently in space (International Space Station, ISS) using the variable speed centrifuges installed in the European modular cultivation system (EMCS). Starting from the real microgravity scenario of the free fall condition, the required acceleration has been produced by means of the use of these dedicated centrifuges. Up to now, this has allowed to get some data on the phototropic behavior of *Arabidopsis* seedlings in these fractional gravity conditions.^25–27^

In this work we aimed at achieving two objectives. First, we established two novel 3D partial gravity paradigms based on the RPM, where fractional gravity was generated by either a hardware configuration (RPM^HW^) or a software- directed partial-*g* RPM (RPM^SW^). Second, we tested and compared the effects of both partial-*g* paradigms using the root meristem as biological model system. For this purpose, we evaluated the status of the meristematic competence (balance between cell proliferation and cell growth) in the roots of young *Arabidopsis thaliana* seedlings. This system had previously been analyzed under real microgravity in the ISS.^28,29^ We have studied the effects, particularly at Moon (0.17 *g*) and Mars (0.38 *g*) partial gravity levels, in comparison with both simulated microgravity and the Earth 1 *g* control.

## Results

### Cell proliferation under partial *g* conditions

**Fig. 1:**
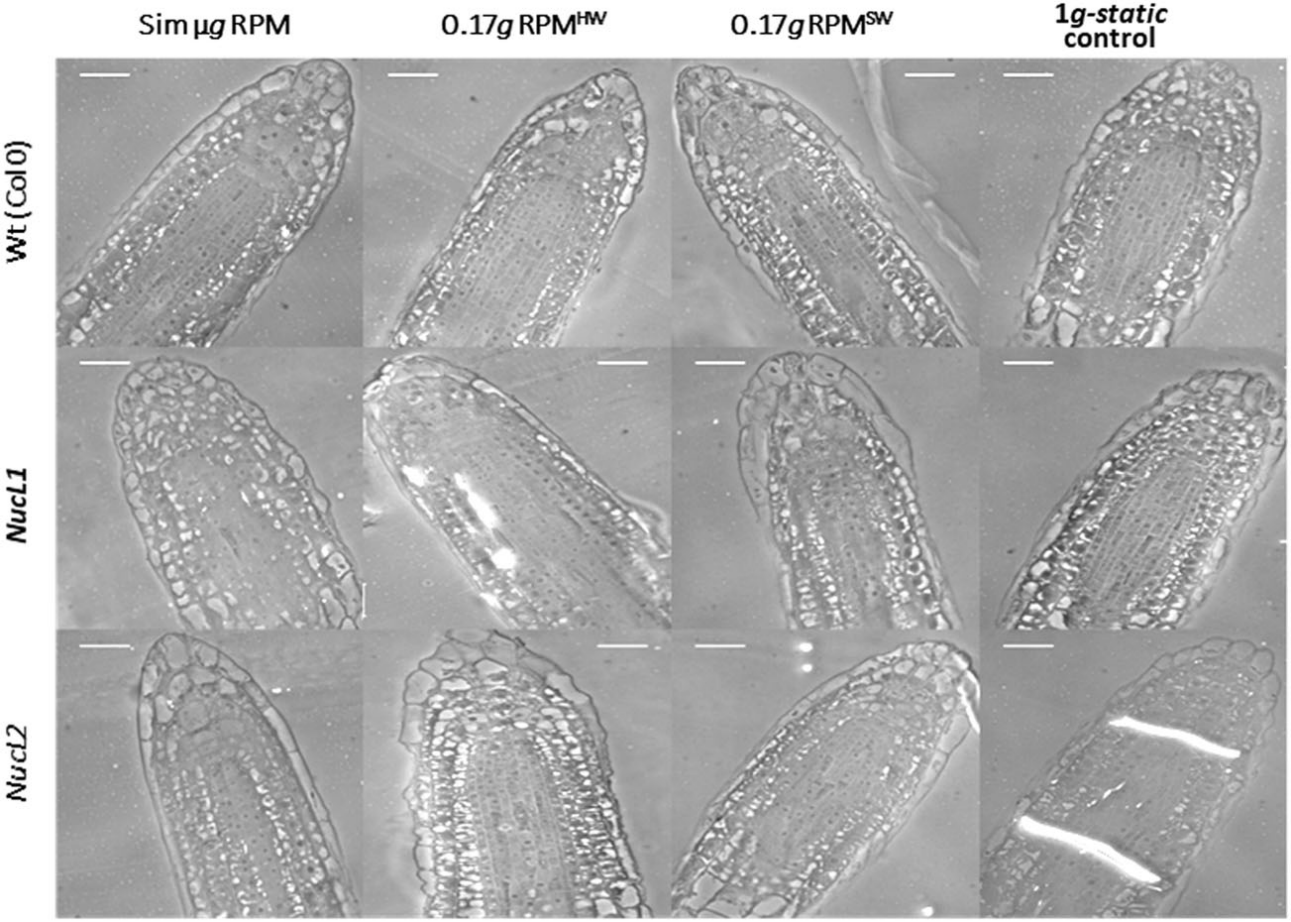
Phase contrast microscopy images from root meristems exposed to simulated microgravity and Moon partial gravity (0.17 *g* RPM^HW^ and RPM^SW^) and 1 *g* static control. Images taken from 2 µm semi-thin sections of fixed and embedded root tips. Identification of the meristematic cell layers and quantitative assessment of the local cell proliferation rate (number of cells per millimeter in each cell row) were performed on them. Images were quantified in Fig. 2. Bars indicate 20 µm

**Fig. 2:**
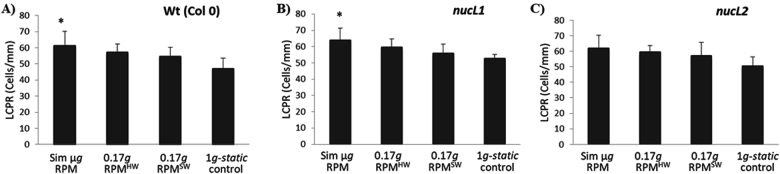
Local cell proliferation rate (LCPR, cells/mm) in roots exposed to simulated microgravity, simulated Moon partial gravity (0.17 *g* RPM^HW^ and RPM^SW^) and 1 *g* static control. Three Arabidopsis lines were used, **a** Wildtype (Col 0), **b** mutant *nucL1*, and **c** mutant *nucL2*. Statistically significant differences vs. 1 *g* static control (*p* < 0.05) indicated with *, average *n* = 4

Differences in the rate of local cell proliferation rate, as an estimation of the proliferative activity, were determined in samples exposed to Moon gravity level using both RPM^HW^ and RPM^SW^ simulators. Three *Arabidopsis thaliana* genotypes were used, namely the wild type (Col-0) and two mutants of the major nucleolar protein nucleolin, corresponding to the two protein variants, *nuc1* and *nuc2*. Nucleolin is a multifunctional protein involved in different cellular processes, most of them related to the regulation of ribosome biogenesis in the nucleolus. Among these processes, chromatin organization and stability, assembly of ribonucleoprotein complexes, and stress response. Consequently, ribosome biogenesis is compromised in these mutants, and, in proliferating cells, this is reflected in the cell growth rate.^30^ While *nuc1* shows a severe phenotype in control conditions (this is the nucleolin variant expressed in the wild type under standard environmental conditions), the *nuc2* mutant shows a phenotype hardly distinguishable from the wild type, because this gene is only expressed either in the *nuc1* mutant, or under stress conditions in wild-type plants.^30,31^ Cell counting was performed on microscopical images of the cortical cylinder layers of the root meristem from samples fixed, embedded and sectioned (Fig. 1). Proliferation as “rate of local cell production” was measured by the number of cells per millimeter in meristematic cell layers.^32^ Consistent with real microgravity results,^28^ this parameter was significantly increased in Col-0 wildtype grown in simulated microgravity (RPM) compared with the static 1 *g* control (Fig. 2a). When partial gravity was analyzed, a similar trend was observed, although the increase in the proliferation rate was smaller and not statistically different. The result was similar in mutant *nucL1* (Fig. 2b), also significant in simulated microgravity conditions compared to static 1 *g*control. A similar trend, although not involving significant differences was observed for the different gravity conditions in the mutant *nucL2* (Fig. 2c). In all cases, the lowest rates of cell proliferation were recorded for the 1 *g*-static control.

### Cell growth under partial *g* conditions

**Fig. 3:**
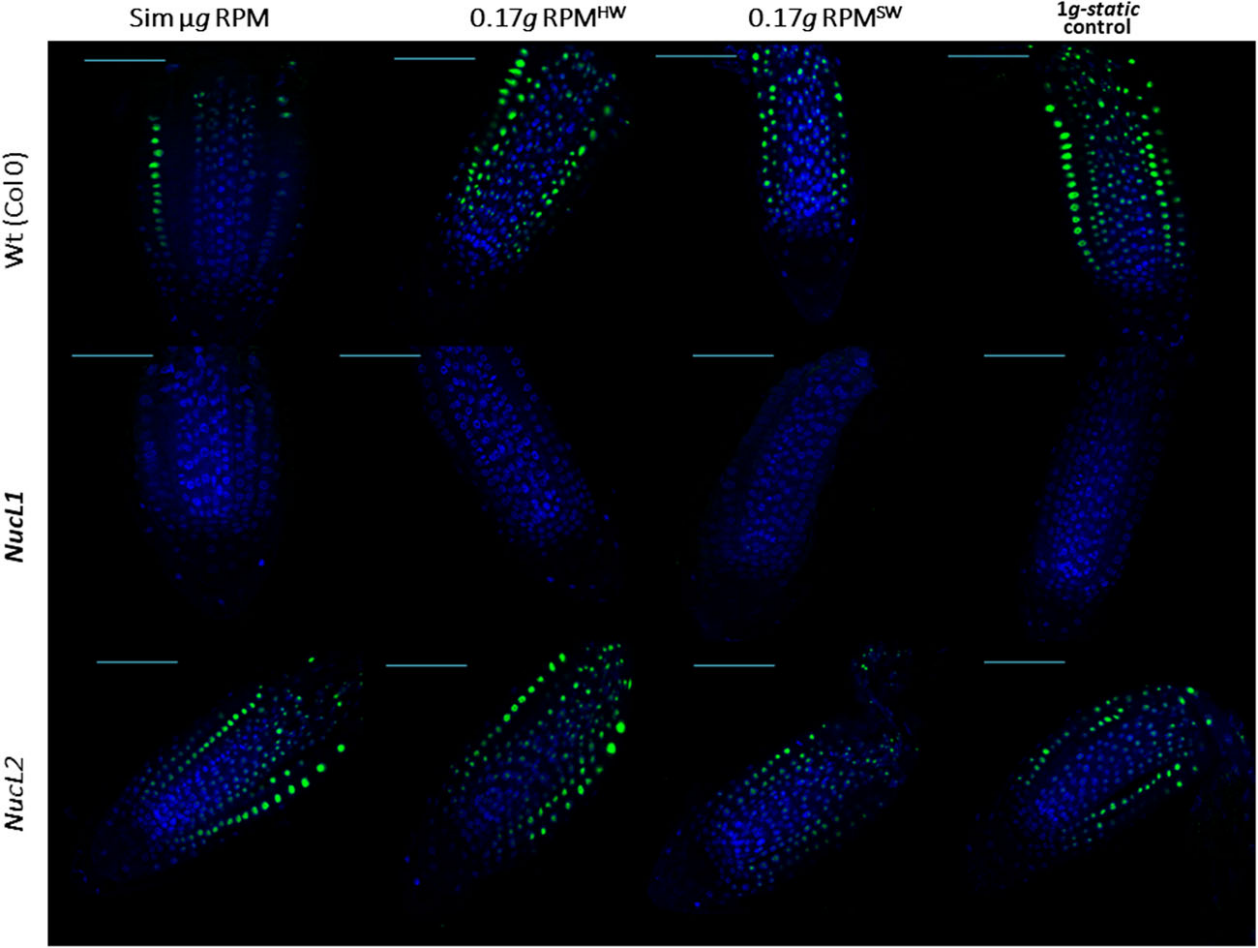
Immunofluorescence images from root meristems exposed to simulated microgravity, simulated Moon partial gravity (0.17 *g*RPM^HW^ and RPM^SW^) and 1 *g* static control. The nucleus of a cell is shown in blue (DAPI signal) while the nucleolus is shown in green (anti-NucL1 signal: note that NucL1 signal is not present in the *nucL1* mutant samples). The size of the nucleolus data represented in Figs. 4 and 5 was measured from such samples. The bars indicate 50 µm

**Fig. 4:**
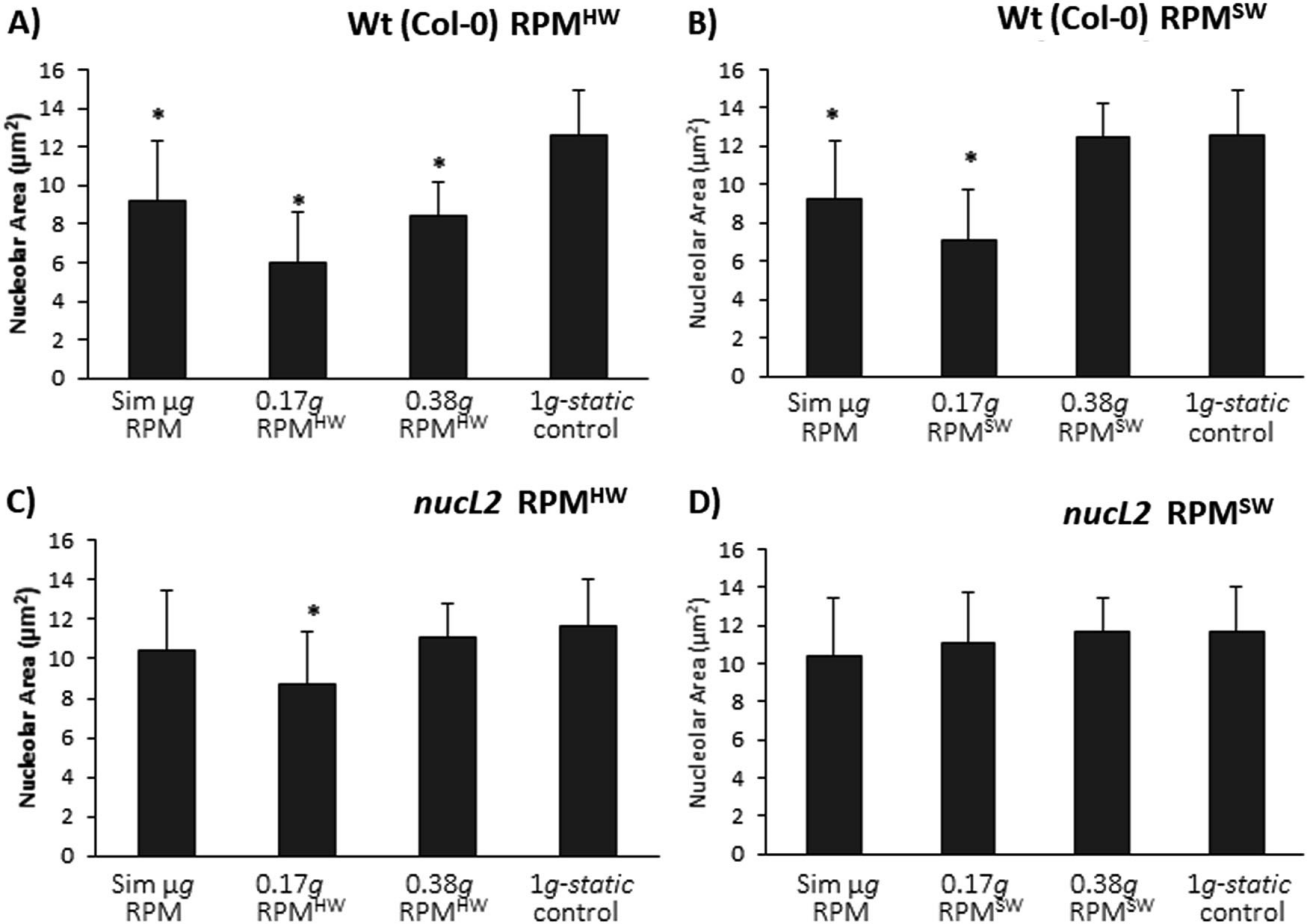
Average size of the nucleolus (area in µm^2^) as determined by the immunohistological detection of NucL1 under simulated microgravity, Moon (0.17 *g* RPM^HW^ and RPM^SW^) and Mars partial gravity (0 .38 *g*RPM^HW^ and RPM^SW^) and 1 *g* static control. **a**,** b** Wildtype line (Col 0). **c**,** d** Mutant *nucL2*. Statistically significant differences (*p* < 0.05) have been indicated with an * *vs*. 1 *g* control, average *n* = 35 in Col 0 and *n* = 73 in *NucL2*

The nucleolar size (as determined by immunohistological detection of Nuc1 protein) was taken as an indicator of the rate of ribosome biogenesis and, indirectly, of cell growth.^33^ This parameter was assessed in samples exposed to Moon gravity level using both RPM^HW^ and RPM^SW^ simulators (Fig. 3). The reduction in the nucleolar size observed under real microgravity^28^ was clearly confirmed in simulated microgravity wild type samples (Fig. 4). In the *nucL2* mutant, a small reduction, without statistical significance can be seen under simulated microgravity. Remarkably, 0.17 *g* partial gravity conditions produce changes relative to 1 *g* control, which are even more significant (smaller nucleoli) than those caused by microgravity, in both the RPM^HW^ (Fig. 4a) and RPM^SW^ (Fig. 4b). For the simulated Mars gravity levels (0.38 *g*), the two methods of partial gravity simulation produced different results. A reduction of the nucleolar size, at levels similar to those shown for microgravity, was observed in the case of the RPM^HW^ version (Fig. 4a) but not in the case of the RPM^SW^, in which the result was similar to 1 *g* static control (Fig. 4b).

When analyzing the same experimental conditions in the mutant *nucL2*, the same trend was noticed, but the differences were smaller and not significant in the simulated microgravity samples. The strong decrease in the RPM^HW^ for Moon gravity was still present and significant (Fig. 4c), but this change was not confirmed in the RPM^SW^ partial *g* simulator, in which none of the samples changed significantly (Fig. 4d).

**Fig. 5:**
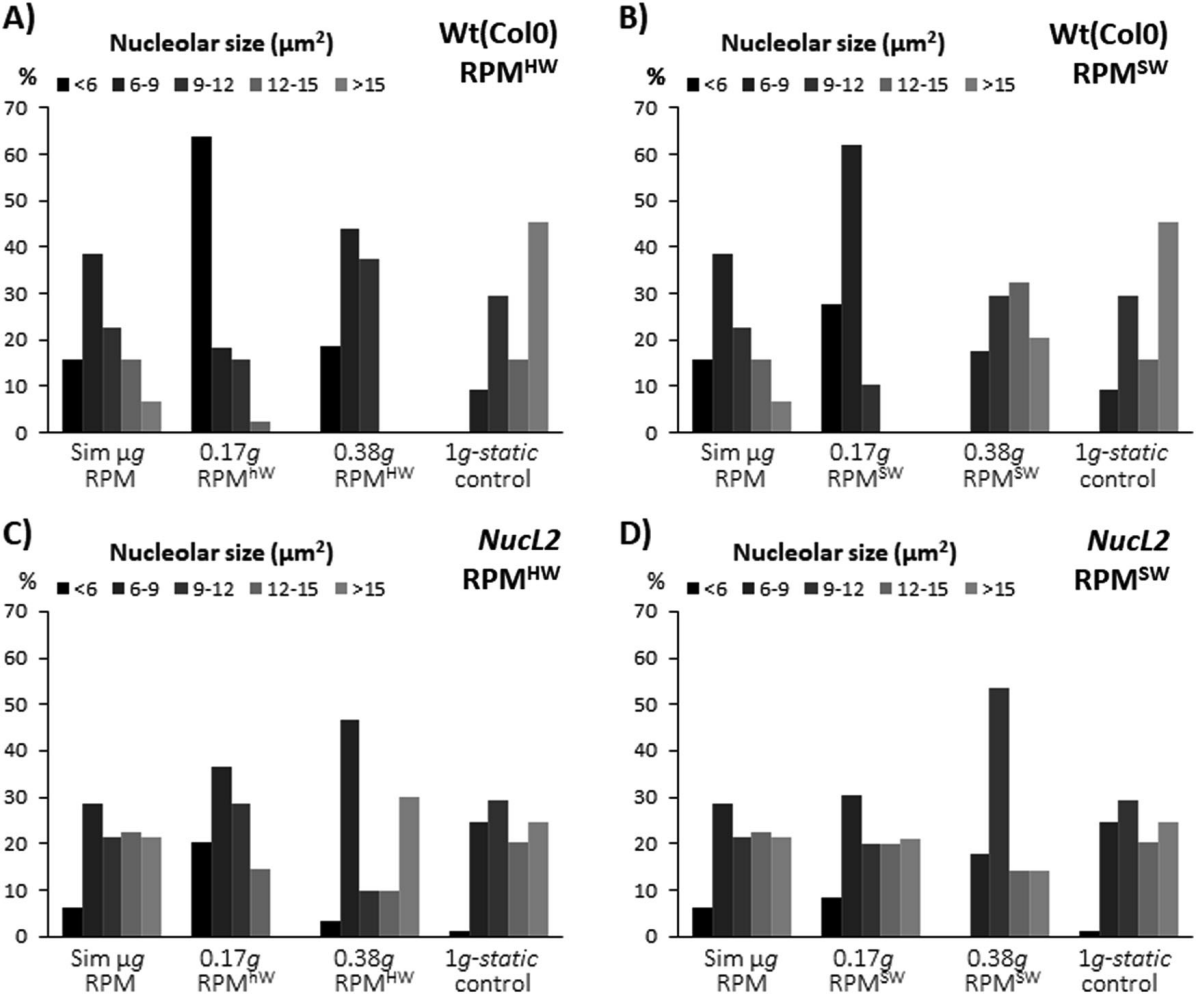
Moon/Mars gravity effect on the distribution of nucleolus subpopulations by size. Percentage of nucleolus falling in each group has been represented (Average area of the nucleolus < 6, 6–9, 9–12, 12–15 or >15 µm^2^) under simulated microgravity, Moon (0.17 *g* RPM^HW^ and RPM^SW^) and Mars partial gravity (0 .38 *g* RPM^HW^ and RPM^SW^) and 1 *g* static control. **a**,** b** Wildtype line (Col 0). **c**,** d** Mutant *nucL2*

To define with higher precision and clarity the effects of simulated partial gravity on the cell growth, we classified nucleoli into five groups according to their size, and applied this classification to all gravitational conditions (Fig. 5). It is very clear that, in the Col-0 wild type plants, the smallest nucleolus group only appears in the simulated microgravity and Moon gravity levels and most of the nucleoli in those samples fall into the two smaller groups. In contrast, the Mars *g*-level and 1 *g* samples have a more balanced distribution of nucleolar sizes (Fig. 5a, b). When the same approach is applied to the mutant *nucL2* samples, a more similar distribution of nucleolar sizes in the various gravitational conditions is observed. Although it is possible to note some smaller nucleoli in the simulated microgravity and Moon *g*-levels, they are more clearly observed in the RPM^HW^ in comparison with the RPM^SW^. The nucleolar size distribution at Mars *g*-level in *NucL2* is more similar to the 1 *g* control than to the simulated microgravity or Moon *g-*levels (Fig. 5c, d), but one can still observe a higher contribution of the two smaller groups of nucleoli to the population for both RPM^HW^ and RPM^SW^ than for the 1 *g* control.

### Cyclin-B1 expression under partial *g* conditions

**Fig. 6:**
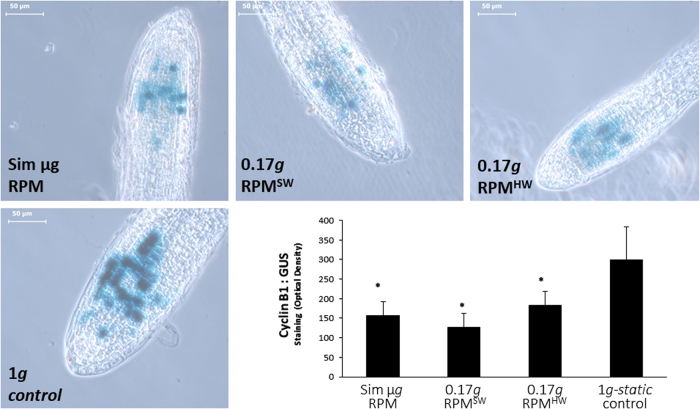
Cyclin-B1 expression in the root meristem determined by the GUS signal in a reporter line. An example of a root tip is provided for simulated microgravity, Moon partial gravity (0.17 *g* RPM^HW^ and RPM^SW^) and 1 *g*static control conditions. The bars indicate 50 µm. In the graph, the Cyclin B1 signal have been quantified as the optical density through all analyzed root tips. Statistically significant differences (*p* < 0.05) have been indicated with * *v*s. 1 *g*, average *n* = 6

Since cell proliferation rate is dependent on cell cycle regulation, we have complemented the analysis with the use of a Col-0 wild type line carrying a Cyclin B1:GUS reporter gene.^34^ Cyclin B1 gene is a known indicator of proliferation, peaking during G2 cell cycle phase,^35^ and it was described as significantly downregulated in real microgravity.^28^ Using this reporter line under simulated partial *g*-load of Moon we found that the effects of this gravity level were similar to those of microgravity. The CycB1 signal was depleted significantly in both RPM^HW^ and RPM^SW^, as strongly as in the simulated microgravity conditions (Fig. 6).

### RPM^HW^ gradient experiment from simulated microgravity to 1 *g* conditions

**Fig. 7:**
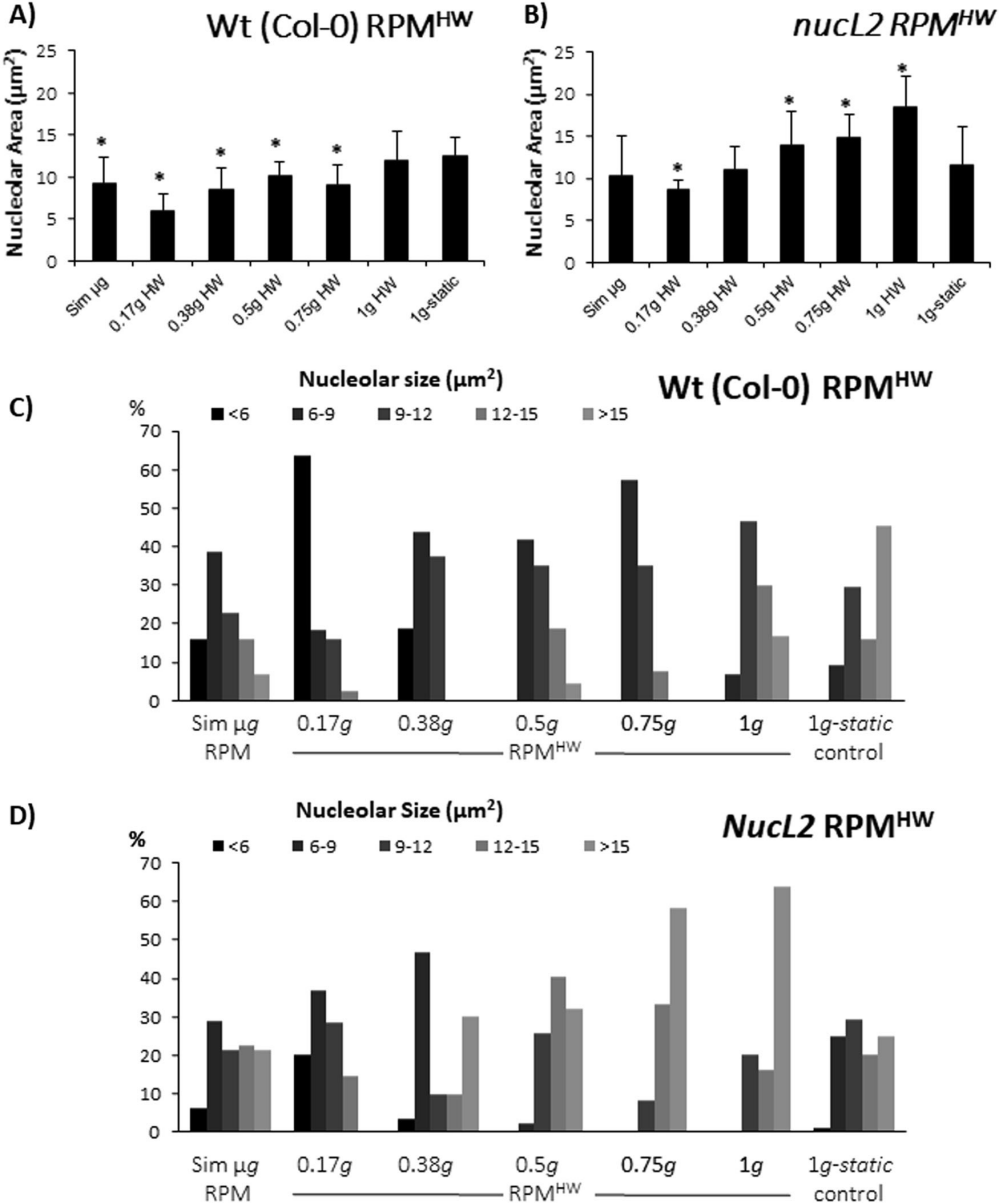
Progressive simulated partial gravity effect on the size and distribution of nucleolus subpopulations. Percentage of nucleolus falling in each group has been represented (Average area of the nucleolus < 6, 6–9, 9–12, 12–15 or >15 µm^2^) in the gradient Hardware partial gravity experiment (RPM^HW^) in comparison with simulated microgravity and 1 *g* static or 1 *g* RPM^HW^ control. **a**, **b** Nucleolus area in Wildtype line (Col 0) and the mutant *nucL2*. Statistically significant differences (*p* < 0.05) have been indicated with * *vs*. 1*g*-static control. Average *n* is 37 in Col 0 and 56 in *NucL2*. **c**, **d** Distribution of nucleolus subpopulations by size in both lines

Finally, we have got a more complete picture of the partial-*g* effects by means of a gradient experiment performed by comparing five different partial gravity levels obtained by using the RPM^HW^ paradigm, together with the simulated microgravity (RPM) and the 1*g-static* control, on two genotypes, namely the wild type (Col-0) and the nucleolin mutant *nucL2* (Fig. 7). In the samples from this experiment the nucleolar size was analyzed. We found that the initial depletion of the nucleolar size at simulated microgravity (even deeper at Moon gravity level) was progressively recovered with the increase of the gravitational load in both strains (Fig. 7a,b). Whereas in the case of Col-0 wildtype plants, the 1 *g* RPM^HW^ condition was nearly the same at the 1*g*-static control, in the case of the *nucL2* mutant, the recovery is even reaching the 1 *g* static control value as early as at the 0.37 *g* level. At the 1 *g* RPM^HW^ position, the average nucleolar size was 50% larger than the static 1 *g* control nucleolar size (Fig. 7b). When applying the approach based on the nucleoli classification by size (Fig. 7c, d) we confirmed that the change in size was progressive and the smaller nucleolar types were substituted by bigger nucleoli according to the *g*-level increase. This effect was particularly visible in the *nucL2* sample at 1 *g* RPM^HW^ conditions, with more than 60% of nucleoli in the largest size group (>15 µm^2^).

## Discussion

In this work, we have analyzed the effects of simulated fractional gravity levels on biological functions, using a plant model system. Very few experiments have been reported up till now on this experimental strategy. The most relevant have consisted on growing plants within a centrifuge operating in the International Space Station.^25–27^

Two ways to simulate a net partial gravity level based on the RPM have been developed and successfully used in this plant biology study, either by the implementation of a hardware (RPM^HW^) or by a software (RPM^SW^) simulation paradigm. The possibility for such a software-controlled partial-*g* RPM was first presented and discussed at ESA conference “Technology for Artificial Gravity and Microgravity Simulation” in December 2007.^36^ A similar system has been published recently,^37^ but there is quite a theoretical difference between the two systems. Different algorithms result in the same partial gravity vector *p*. The simplest is pointing an experiment in the gravity direction for a fraction *q* of the time and in the antigravity direction for a fraction 1−*q* of the time with *q* = (*p* + 1) / 2. However, the partial gravity vector *p* is only one of the properties of the algorithm. Other properties are the distribution of the gravity vector, speed and depth of the convergence, accelerations of the experiment, and velocities and accelerations of the motors that may affect the outcome of an experiment. For the theoretical zero gravity our algorithm converges to a uniform distribution over a sphere while other algorithms from literature converge to non-uniform distributions.^38,39^ Similarly, for partial gravity our algorithm converges to a uniform distribution over a spheroid while other algorithms (i.e., Benavides et al.^37^) converge to distributions that are less uniform. The definition of these properties and the comparison of different algorithms with respect to these properties may be the topic of a separate, more mathematical, future paper.

Regarding the RPM^HW^, although theoretically this concept could also be implemented in the more widely used desktop RPMs, it is preferred to have a larger diameter system in order to diminish the *g* gradient and inertial shear effects associated to short radii centrifuges.^40,41^ However, one should take note of increased fluid movements if such a system is used for, e.g., cell cultures experiments.^42–44^ For regular RPM studies it is important to place the sample as close as possible to the center of rotation in order to minimize residual *g* artifacts.^20,45^ However, in the partial-*g* RPM^HW^ we want to have a rotating disk as large as possible to minimize centrifuge associated artifacts like the aforementioned sample gravity gradients. For the 200 mm radius of the current centrifuge platter and an RPM standard maximum rotation speed of 60° s^−1^ results in a 0.02 residual *g*. This 0.02 *g* is superimposed onto the partial gravity level generated by the centrifuge. Although this might be a significant error when one wants to simulate microgravity, it is a relatively limited error of ~5% compared to an, e.g., 0.38 *g* Mars simulation. Consequently, the RPM^SW^ may be used below the Mars *g*-level while the RPM^HW^ should be preferentially used beyond that *g*-level, or samples should be placed more closely to the center of rotation or maximum the acceleration setting of the RPM could be reduced from the current 60° s^−1^, in order to reduce this artifact.

Both partial gravity paradigms are based on two completely different concepts. Where the RPM^SW^ is a time/orientation-based partial gravity generation, the RPM^HW^ is a system more based on centripetal acceleration. The best simulation paradigm may be different for any given biological model system and more research is needed to explore these paradigms.

Our plant biology results clearly show that the exposure to simulated fractional gravity conditions with a magnitude of 0.17 *g* Moon gravity produce substantial alterations in root meristematic cell growth and proliferation parameters and consequently lead to the disruption of the meristematic competence during early plant development. We found an increase in the local cell proliferation rate and, in contrast, we found a decrease in the nucleolar size detected by nucleolin immunolabeling, indicating a depletion in the rate of pre-rRNA transcription and processing,^46^ and, subsequently, in protein synthesis. These results are consistent with previous observations reported by our laboratories in real microgravity experiments^28^ and in ground-based facilities.^41,47^ Whereas most of the alterations found at the simulated Moon gravity level are quantitatively even more intense than those observed in simulated microgravity, this does not occur at the second level of fractional gravity that we tested, i.e., 0.38 Mars *g*. In this case, the alterations are mild, usually not clearly different from the 1 *g* static control condition. This finding is especially important in the determination of the magnitude of the gravity vector that is the threshold for the gravity sensing systems and mechanisms of the plant. According to our results, and based on the response of the root meristematic cells, this threshold can be estimated intermediate between the Moon gravity (0.17 *g*) and the Mars gravity (0.38 *g*). The consequence is that plants grown under Moon gravity would suffer similar meristematic competence lost as those grown under microgravity, but in plants grown under Mars gravity meristems cell cycle and cell growth balance will be similar to those observed under Earth gravity. The preliminary results obtained by our laboratories in a recent ISS experiment, are in support of this proposal. In that experiment, seedlings grown in space under a gravity level of 0.3 *g*, obtained with a centrifuge installed in the EMCS facility, showed their roots oriented along the gravity vector. This means that 0.3 *g* is sufficient to be sensed by the root such that a gravitropic response is effectively triggered in seedlings grown. In other words, 0.3 *g* appears to be above the threshold of gravity sensing by the specialized systems of the plant. If these findings were confirmed by further experiments testing the degree of alteration of other physiological processes of the plant, the consequences for space exploration would be highly relevant, affecting the strategy of plant culture on the Moon, and likely less problematic on Mars.

The cellular effects observed in samples grown under simulated Moon partial gravity indicate the existence of alterations in the regulation of the cell cycle, leading to a premature cell division, before the cell has reached the proper size as required at 1 *g* control conditions. Checkpoints for cell size occur indeed just in the G2 stage and the G2-mitosis transition^48^ and it is known that the major part of ribosome production (and consequently, of cell growth) in cycling cells occurs in G2 as a preparation for mitosis.^49^ Hence, a shortening of the G2 phase could result in a reduction in a cell growth and in an acceleration of cell division, producing a greater number of smaller cells. This interpretation is supported by previous experiments in real and simulated microgravity, showing that the amount of nucleolin was reduced.^28^ A shorter duration of G2 would also give account of the CycB1:GUS results of this paper, in which the expression of this gene is reduced, under simulated Moon conditions, with respect to the 1 g static control. Since CycB1 is mostly expressed at G2, its downregulation may be linked to a reduced number of G2 cells. In fact our experiments performed in cell cultures under simulated microgravity have recently shown that the cell cycle progression is faster under simulated microgravity conditions, particularly through a reduction in the duration of the G2 phase promoting a quicker entry into cell division.

Other studies on partial-*g* simulation have used magnetic levitation,^50^ or the combination of clinostats and parabolic flight.^51^ Cell cultures used in these studies were analyzed by transcriptomic methods. Although those cell culture studies were constrained with the limitations of the short-term exposures (analyzing the initial response to a change in *g* level), interference with the magnetic field or successive parabolas on the actual gravitational load those cultures experienced, they were suggesting the importance to verify microgravity results using partial *g* levels. For example, after 3 h magnetic levitation, a greater disturbance of the transcriptional status was observed under 0.1 g* than at the nominal 0 g* levitation conditions.^50^ This is in agreement with the results shown in this paper, reporting stronger effects of the simulated Moon gravity with respect to simulated microgravity.

The RPM^HW^ paradigm provides the possibility of running a sort of internal control where the centrifuge can be set to generate 1 *g* allowing a comparison with the 1 *g* static control samples. This would allow identification of effects of centrifugation and RPM artifacts in the parameters studied. A striking result obtained in the gradient RPM^HW^ experiment (Fig. 7) is that, in the case of the wild type samples, the values obtained at 1 *g* are similar whether this magnitude of *g* was obtained in the static control, or using the centrifuge on the RPM running at 1 *g*. However, this does not occur in the *nucL2* mutant, in which the two values are significantly divergent. This is an interesting result, since the mutant is defective in the nucleolin-2 variant, a protein known to play a role in the stress response.^52^ This may explain the variations, regarding the size of the nucleolus, of the response to gravitational changes (no significant variations between the simulated microgravity or the Moon *g*-level with respect to the 1 *g-*static control) and the response to other types of mechanical stimuli (significant variations between the 1 *g* RPM^HW^ and the 1*g*-static control). However, it cannot be excluded that these differences are due to the mechanical stress or small residual vibrations due to centrifugation, in the case of hardware simulation. A similar suggestion was made in comparable plant experiment using hypergravity (2 *g*) in the large diameter centrifuge, where differences in nucleolar size were found between the 1 *g* rotational control and 1 *g* static control.^53^

The result for the wild-type (Col-0) seems ideal in that there is no significant differences between 1*g*- RPM^HW^ and 1 *g* static control, but a different distribution is seen in the nucL2 mutant group. Also in this case, more research, including the use of various *g*-levels up to 2 *g*, is needed to further explore these differences.

The results shown here are promising and encourage the future exploration of both paradigms in more detail and using other biological systems and assays. However, important verification of the actual responses should be performed with dedicated experiments on board the ISS, using facilities as the EMCS, the Cell Biology Experiment Facility, Kubik or Biolab. The centrifuges in these facilities could be set to generate various partial *g* levels and the results obtained should be compared with the ground-based simulations. Such studies should not be confined to exploring effect on plants, but also single cells and small animals should be used. Although clinostats (2D and 3D versions) as well as partial-gravity simulators can contribute significantly to our understanding of mechanobiology, e.g., cell physiology and or plant tropisms, one should always realize that these devices are only simulating the low gravity conditions.^45,54^ The real effects should be tested either in a proper centrifuge experiment on board a free falling spacecraft or actually on the surfaces of Moon and Mars.

## Materials and methods

### Set up of partial *g* systems: HW and SW simulation strategies

**Fig. 8:**
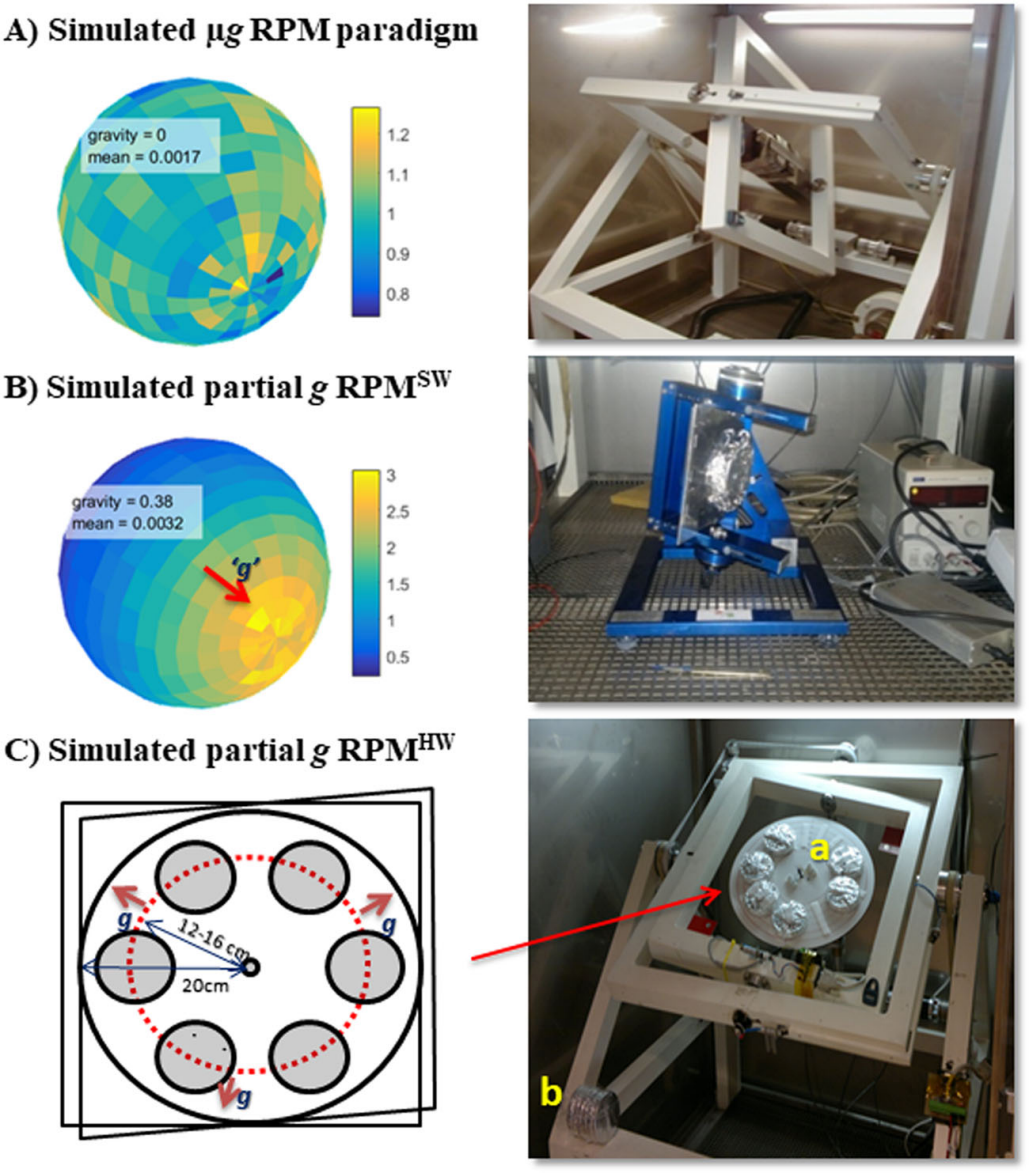
Different RPM paradigms for partial gravity simulations. The Random Position Machine (RPM) can be used to simulate both, microgravity (µ*g*) and partial gravity (at, e.g., Moon (0.17 *g*) or Mars (0.38 *g*) levels). **a** Large size RPM with two independently driven perpendicular frames located inside a large incubator. Real random mode with 0 eccentricity allows full randomization of the gravity vector (simulated microgravity); gravity = 0. Mean is the mean error between the desired and the actual partial gravity factor. Experimental samples placed in the very center of the RPM increases the quality of the simulated microgravity. **b** Desktop RPM (RPM version 2.0: Airbus Defense and Space, the Netherlands B.V., Leiden, the Netherlands) applicable to be used in a standard incubator using dedicated software (RPM^SW^) controlling the speed and axes angles with respect to the Earth’s gravity vector. In this example the orientation distribution over a spheroid is with an eccentricity of 0.53, corresponding to a Mars gravity level of 0.38 *g*. Mean is the mean error between the desired and the actual partial gravity factor. See also Supplementary Information for more details. **c** A large size RPM adapted for simulating partial gravity using the hardware paradigm (RPM^HW^). It consists of the RPM as in A providing the simulated µ*g* environment. Inside, a centrifuge is accommodated with a maximum radius of 20 cm. Samples should be properly placed to produce an exact partial gravity level (see Table 1). The scale bars represent the normalized density where 1 is uniform density. The picture shows experimental samples located on the centrifuge (**a**) and the 1 *g*static control samples which are placed on the RPM frame in the same incubator (**b**). All instruments are located at the technology center of the European Space Agency (ESA) Lis Laboratory (ESTEC-TEC-MMG), Noordwijk, the Netherlands

Two ways to induce a net simulated partial gravity level, similar to Moon (0.17 *g*) or Mars (0.38 *g*), have been developed using the RPM (Fig. 8a). The first was based on the partial neutralization of 1 *g* by means of the software which drives the time averaging specific orientation of the samples (RPM-software, RPM^SW^, Fig. 8b). The second approach was based on neutralizing the effect of gravity first (regular RPM paradigm), and on top of this apply the partial gravity with a built-on centrifuge (RPM-hardware, RPM^HW^, Fig. 8c and Supplementary Information Figure S1).

The effect of the software modification applied in the RPM^SW^ procedure is to change the uniform distribution of points on a sphere resulting in a real surface average for simulated micro-*g* existing in a regular RPM, by means of an algorithm which directs points over a prolate spheroid. One of the focal points of the spheroid is placed in the rotation center of the RPM. The larger the eccentricity of the spheroid, the greater the deviation from the microgravity simulation, resulting in a partial-*g* simulation. In other words: by travelling over the ellipsoid with the same speed visiting randomly all sections of the surface, the residence time at one side of one of the spheroid focal points is longer than the time at the other focal point. Within the random motion, the specimen spends more time in one orientation/direction, resulting in an average *g* level in between the theoretical 0 and 1 *g* depending on the path settings. The distribution of the gravity vector over the spheroid with an eccentricity of 0 (a perfect sphere), corresponds to a partial gravity vector of 0, is shown in Fig. 8a. Distribution of the gravity vector over a spheroid with an eccentricity of 0.53 corresponds to a partial gravity vector of 0.38 *g*, or Mars *g* (Fig. 8b). After 1 h of rotation the errors of the mean gravity have converged to 0.0017 and 0.0032, for simulated microgravity and Mars *g*, respectively. (See also Supplementary Information for more details).

The RPM^HW^ is based on the premise that a regular RPM simulates near weightlessness. When we take this as the base gravity level one can apply centrifugation to generate any *g* level we need. The current system is capable to generate *g* levels from nearly zero up to two *g*. For this RPM^HW^ we made use of a large size RPM (inner frame 500 × 500 mm) fitted with a rotating platform of 400 mm diameter. The platform is driven by a regular electromotor speed-controlled by a standard tachometer. Power and data to the platform is provided by a 12 channel slipring. For operational flexibility samples are fixed to the rotating platter by Velcro® (Fig. 8c). Table 1, lists the actual settings used to simulate five partial gravity conditions including Moon and Mars scenarios.

**Table 1:** Setting for RPM^HW^ and RPM^SW^ partial gravity paradigms

Simulated Condition	Acceleration (*g* level)	RPM^HW^		RPM^SW^
		Angular speed (rpm)	Radius (cm)	Eccentricity
Moon	0.17	36	12	0.25
Mars	0.38	53	12	0.53
1/2 *g*-Earth	0.50	53	16	0.66
3/4 *g*-Earth	0.75	75	12	0.87
1 *g* HW control	1.00	75	16	—

For the RPM^HW^ the internal centrifuge settings include three angular speeds (in revolutions per minute) combined with two distances from the center of rotation (radius in cm) to provide the desired fractional *g* level. In the column on the right the RPM^SW^ parameters (eccentricity) are shown for the same gravitational levels

### Set up plant experiment

In this experiment four *Arabidopsis thaliana* ecotype “Columbia” lines were used: (1) the wildtype strain (Col-0). (2) Two mutants of the two variants of the major nucleolar protein nucleolin. This protein is a chief regulator of ribosome biogenesis, whose variations affect cell growth rates.^31^ The mutant *nucL1* is defective in the protein variant AtNucL1, clone 1.2, and the mutant *nucL2*, is defective in the protein variant AtNucL2, clone 2.2. Both mutants were kindly provided by Dr. J. Sáez-Vásquez (CNRS/Univ. Perpignan, France). (3) A line carrying a CycB1:GUS reporter gene construction to detect the expression of the cyclin B1 gene and to evaluate the cell cycle progression during G2/M phase.^55^ This mutant was kindly supplied by Dr. E. Carnero-Diaz (UPMC, Paris, France).

Seeds were sterilized in 70% (v/v) ethanol and 1% (v/v) Triton X-100 for 4 min and washed with 95% ethanol (v/v) (2 times, 1 min). Then, 25 seeds were placed in an equi-gravity line (i.e., curve) at 12 or 16 cm from the center of the centrifuge (Fig. 8c, as described in Table 1) on 9 cm diameter Petri dishes containing 0,8% agar with MS (Murashige and Skoog’s, Duchefa) plant culture medium. They were exposed to pretreatment at 4 °C for 2 days in order to produce a quick and synchronous germination just after loading them into the RPM.

The plates were placed at four altered gravity positions in each experiment:

A. Sim µ*g*: simulated microgravity conditions in standard RPM running the real random mode (nominally µ*g*). Settings: maximum acceleration 60°/s, random direction and random interval.
B. RPM^HW^ fractional gravity, by means of exposing the samples to an RPM environment (nominally µ*g*) plus a centrifugation to provide the desired *g* level (RPM settings same as in A, see also Table 1).
C. RPM^SW^ fractional gravity: using a modified averaged algorithm, the RPM will run a custom program that favors the samples to expend more time in bottom positions *vs*. top positions in the 3D spheroid to generate a residual *g* level of interest.
D. Static 1 *g* control: samples in the same incubator and attached to the static structure of the RPM.

Seeds were allowed to germinate within the simulation facilities by incubating them at 22 °C. After 4 days growing in dark conditions, samples were photographed and quickly recovered from the dishes. *Arabidopsis* seedlings were transferred into the fixative solutions, either 3% (v/v) glutaraldehyde, for structural microscopical analyses, or 4% (v/v) paraformaldehyde, for immunofluorescence. CycB1:GUS seedlings were dehydrated by immersion in cold acetone.

### Sample processing for structural analysis and quantification

Samples for microscopical analyses remained in 3% glutaraldehyde for 3 days at 4 °C, and were then transferred to PBS for washing (3 times, 10 min) and dehydrated in an ethanol series. Finally, they were subjected to methylation-acetylation and embedded in LR White resin (London Resin Co., UK).

From resin-embedded materials, semi-thin sections, 2 µm thick, were obtained and observed unstained under a Leica DM2500 microscope equipped with phase contrast. Images were recorded with a Leica DFC320 CCD camera. Quantitative measurements (number of cells per mm in root cell rows) were carried out on digital images using the quantitation software ImageJ.

### Sample processing for immunofluorescence and quantitation

Seedlings were fixed in 4% paraformaldehyde for 3 days at 4 °C, washed three times 5 min with PBS and incubated in cell-wall-digestion solution (2% cellulase, 1% pectinase, 0.005% macerozyme, 0.4% mannitol, 10% glycerol, 0.2% Triton-X100 in PBS) for 45 min at 37 °C. After incubation, the digestion solution was removed, seedlings were washed with PBS + 10% glycerol + 0.2% Triton-X100 (3 times, 5 min) and excised apical parts of roots were placed onto glass slides covered with L-polylysine. Samples were then dehydrated with 100% methanol for 15 min at −20 °C and air dried. Slides were washed with PBS (twice, 5 min), pretreated with blocking buffer (PBS + 0.05% Tween + 2% BSA) for 30 min at room temperature and incubated overnight (37 °C) in a humidified atmosphere with a rabbit antibody raised against AtNuc-L1^31^(diluted (1:1000) in blocking buffer. Following incubation, slides were washed 3 times with PBS and incubated for 3 h (37 °C) with a second antibody (anti-rabbit IgG coupled with ALEXA 488), diluted (1:100) in blocking buffer. Then, samples were washed with PBS (thrice, 5 min) and nuclear DNA was stained with 4´,6-diamidino-2-phenylindole (DAPI). Finally, slides were washed with PBS and distilled water (twice, 5 min) and mounted with an anti-fading agent (DABCO 33-LV; Aldrich Chemical). Observations were made with a confocal laser microscope (Leica SP 5). Nucleolar size (area immunostained for nucleolin) was measured using the quantitation software ImageJ.

### Sample processing for CycB1:GUS reaction

Samples were incubated in acetone at −20 °C for 3–4 days. Then, samples were washed with 100 mM phosphate buffer and the GUS signal was revealed by enzymatic reaction (5 mM potassium ferrocyanide and ferricyanide, 100 mM phosphate buffer and 40 mM X-Glc A) in the dark. Seedlings were washed and mounted in 8 × 8 mm well slides and observed under a Leica DM2500 microscope. Images were recorded with a Leica DFC320 CCD camera and processed with Image J software. Integrated optical density was calculated as the product of the stained area by the optical density in the blue light spectrum. An unstained zone of the root tip was used as blank.

### Statistical analyses methodology

Measurements of cell parameters were performed on root meristematic cells belonging to the epidermal, cortical and endodermal cell layers of the root. Statistical analysis of data was performed using SPSS 17.0 software. Description of quantitative variables was performed using mean and standard deviation values. Mean values were compared using the Student *t*-test for independent samples; differences were considered significant for a bilateral probability value, *p*, lower than 0.05.

## Electronic supplementary material

Supplemenentary Information
