# Supplementary material for: Novel, Moon and Mars, partial gravity simulation paradigms and their effects on the balance between cell growth and cell proliferation during early plant development
Source: NPJ Microgravity. 2018 Apr 4;4:9. doi: 10.1038/s41526-018-0041-4 (PMC5884789; doi:10.1038/s41526-018-0041-4)
Supplement: Supplementary file 1 — Supplemenentary Information [file 41526_2018_41_MOESM1_ESM.docx]

**Supplementary Information**

**Background of the Software RPM**

The RPM is a mechanism that consist of two perpendicular axed frames driven by motors that continually rotate an experiment frame relative to the world frame (Figures 1 and S1). Or since the gravity direction is fixed in the world frame, it continually rotates the gravity direction relative to the experimental frame ergo the sample. The gravity vector acceleration can be averaged over time resulting in a certain mean gravity ranging from the theoretical 0 g to 1 g where 1 g is the Earth gravity acceleration of 9.81 m/s^2^. If the mean gravity is close to 0 g this is called zero gravity or better microgravity, in order to take into account small deviations such as distance from the center of rotation (see e.g. ^1,2^). If the mean gravity is between 0 and 1 g it is referred to as partial gravity or hypo-gravity. Note that the experiment must be placed in the center of the experimental frames to prevent it from being exposed to possibly significant centrifugal acceleration ^1,3^.


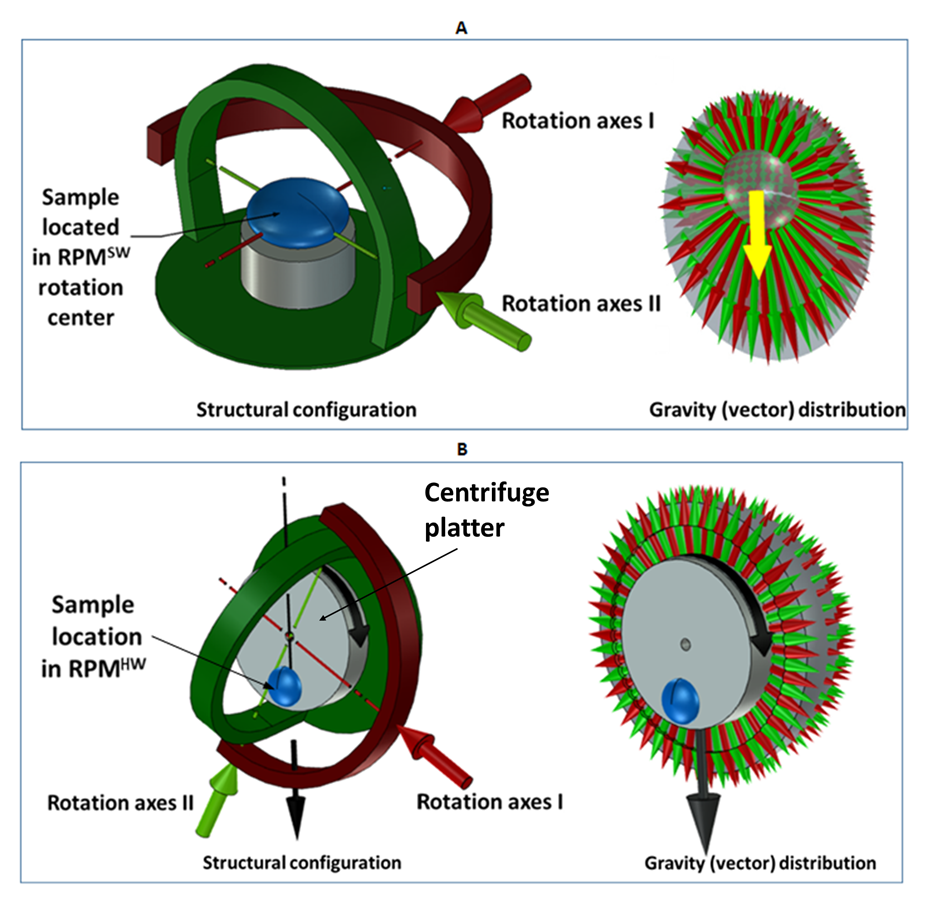


*Figure S1:* *Set up of partial g simulation systems. A: Cartoon of the software directed simulated partial gravity Random Positioning Machine (RPM^SW^). Left the hardware / axes configuration. Right: Software directing the motion of this RPM can be set to have a certain degree of preference along the Earth gravity vector. Resultant g represented by yellow arrow. B: The hardware directed simulated partial gravity RPM (RPM^HW^). Left the hardware configuration consisting of a regular (large size) RPM where the partial g is generated by a centrifuge (grey). Right the resultant g-load depicted by the black arrow pointing from the rotation axis of the centrifuge outwards.*

**Kinematics**

The joint space of the mechanism is the set of motor angles (q1, q2) and forms a 2-plane. The task space is the set of gravity directions relative to the experiment and forms a sphere (Figure S2A). The sphere is embedded in 3-space and can be parametrized by the three components of the gravity vector (g1, g2, g3). The function from the motor angles to the gravity direction is called the forward kinematics and is given by:

$${gravity\left( t \right)=forward \left( q \right)=\left[ \begin{matrix} cos\left( q_{1} \right)cos\left( q_{2} \right) \\ cos\left( q_{1} \right)sin\left( q_{2} \right) \\ sin\left( q_{1} \right) \end{matrix} \right]}$$

where the motor angles can be identified with the latitude and longitude of a sphere. The inverse function from the gravity direction to the motor angles is called the inverse kinematics.

The motor angles follow a defined path in joint space q(t) that results in a certain gravity direction path in task space g(t). The goal is to set a motor path q(t) such that the gravity path g(t) has the desirable properties. In the following two such desirable properties for g(t) will be proposed and one algorithm for choosing a motor path q(t) that has these properties. First they will be discussed in the context of microgravity and later they will be generalized to partial gravity.

**Zero mean**

The gravity vector g(t) can be averaged over time resulting in a certain mean gravity:

$$mean\left( gravity\left( t \right) \right)=\frac{1}{t}\int gravity\left( t \right)dt$$

The first and most obvious desirable property is that the mean converges to zero or (0, 0, 0). Most algorithms from literature have this property ^4,5^. For example also a simple clinostat has it. However, for some samples this may not be sufficient. Note that the rate of convergence must be fast compared to the relevant time constants of the experiment. For example the perception time for plants is in the order of seconds ^6^, for the single cell organism *Euglena gracilis*, a similar time order of around five seconds has been reported ^7^.

**Uniform distribution**

The space of al gravity directions provides a sphere. The gravity path g(t) walks over this sphere and visits different points. The relative times that it spends in the neighborhoods of different points result in a certain distribution over the sphere. If the path visits all points equally the path converges to a uniform distribution (Figure S2A), and if it visits some points more than others the path converges to a non-uniform distribution. A simple 2D clinostat for example only visits 1 great circle on the sphere (which can be identified with the equator of the sphere), and therefore results in a very non-uniform distribution. Different distributions can result in same mean gravity. For example both the uniform distribution and the clinostat distribution result in the same zero mean.

Certain experiments may be more sensitive to gravity in some directions than in other directions, and are therefore sensitive to the distribution of the gravity vector. Such experiments would result in different outcomes for different distributions. Note that such experiments are also sensitive to how the experiment is fixed in the experiment frame because the distribution is relative to the experiment frame.

The only distribution that has no bias in certain directions is the uniform distribution. Therefore the second desirable property of a gravity path is that the distribution converges to a uniform distribution. Note that also here the rate of convergence must preferably be less compared to the relative time constants of the experiment.

A good measure for the uniformity is the standard deviation of the density of the distribution over surface of the sphere. Here density (t, q) is the amount of time the gravity direction has spent in a neighborhood of f(q).

$$standard deviation\left( density\left( q,t \right) \right)=\sqrt{\frac{1}{4\pi t}\iint\left( density\left( q,t \right)-\frac{t}{4\pi} \right)^{2}cos\left( q_{1} \right)dq_{1}q_{2}}$$

Most algorithms from literature (for example figure 4 of Wuest *et al*.^4^) including the algorithm for the first generation of the RPM ^2^ result in a uniform distribution in the latitude – longitude plane. However, when this plane is folded around the sphere the density at the poles becomes much higher than at the equator. This is similar to a globe where the density of meridians at the poles is higher than at the equator. Although the first property (zero mean) is met, the second property (uniform distribution) is not met.

**Random walk**

The gravity path that is proposed consists of a random walk over the surface of the sphere. The random walk consists of a sequence of steps and turns. The steps are geodesic curves of a certain length which on the surface of a sphere correspond to pieces of great circles. The turns are uniformly distributed between plus or minus a defined maximum angle. It can be proven that such a random walk converges to a uniform distribution over the sphere ^8^.


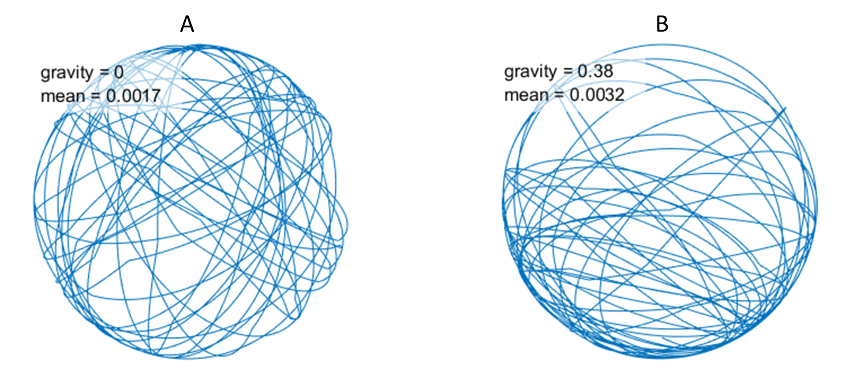


*Figure S2:* *The beginning of a path of the gravity vector over the sphere for zero gravity (on the left) and for Mars (on the right). It can be seen that on the left the trajectory is distributed uniformly over the sphere, while on the right it trajectory spends more time at the bottom than at the top, resulting in a partial gravity factor of 0.38. Mean in the figure is the mean error between the desired and the actual partial gravity factor after running for 1 hour.*

**Generalization to partial gravity**

The algorithm for zero gravity (random walk over a sphere) can be generalized to partial gravity by introducing a prolate spheroid. A prolate spheroid is an ellipse that is rotated around its major axis and has the shape of a rugby ball (see Figures 1B and S3). It has two focal points. One of the focal points is fixed in the center of the sphere of gravity directions, and the other focal point is moved in the direction of the desired partial gravity. Now instead of performing a random walk over the sphere a geodesic random walk over the spheroid is performed. The resulting random walk will converge to a uniform distribution over the spheroid. The proof from ^8^ still holds and this random walk is then projected from the spheroid onto the sphere.

Because a relatively large part of the surface of the spheroid is on one side of the sphere and a relatively small part on the other side (Figure S2B), the gravity direction spends more time on that side of the sphere. So if the gravity vector is averaged over time, it converges to a certain non-zero partial gravity. By varying the ellipticity of the spheroid from 0 to 1 the simulated partial gravity is varied from 0 to 1.

The two desirable properties can be generalized to partial gravity as follows. For the first property (zero mean) instead of looking at the norm of the mean gravity we look at the norm of the difference between the mean gravity and the desired partial gravity. For the second property (uniform density), instead of looking at the standard deviation of the density of the distribution over the sphere, we look at the distribution over the spheroid.


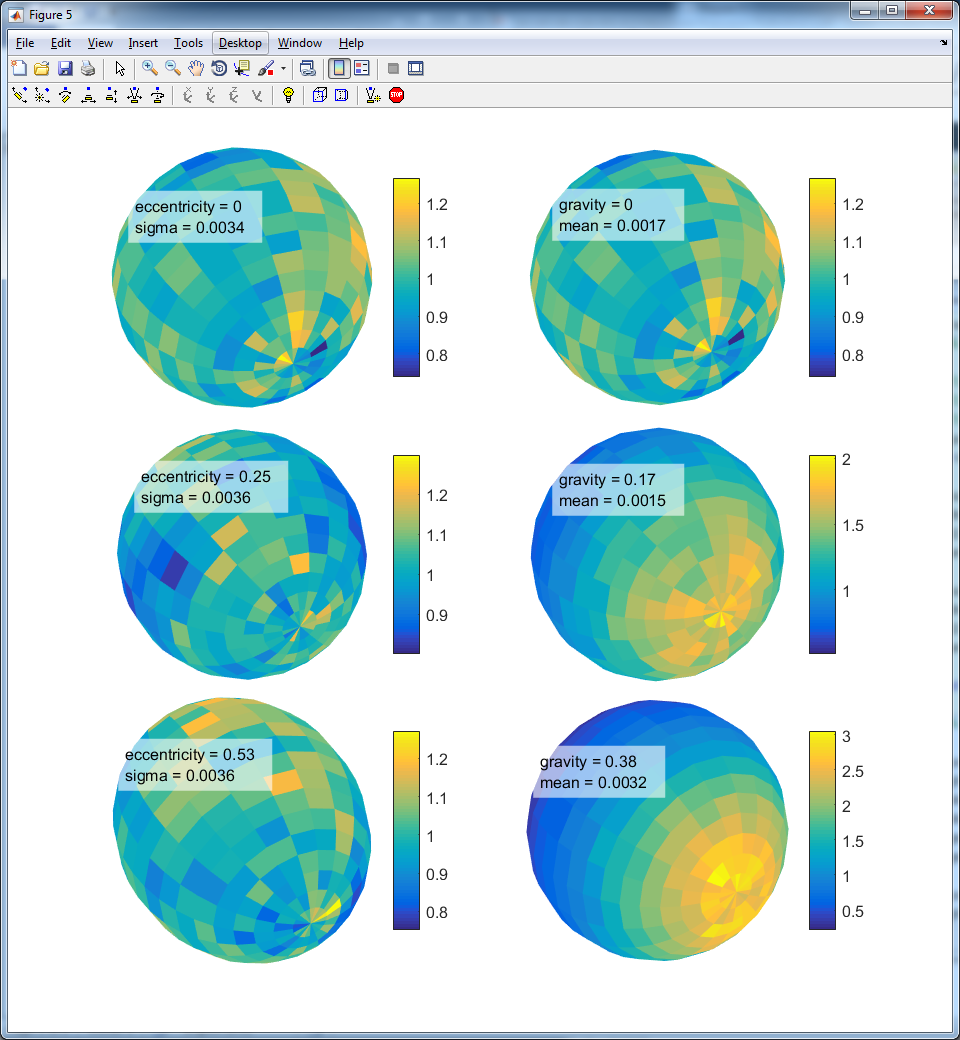


*Figure S3: Distribution of the path over a spheroid (on the left) and resulting distribution of the gravity vector over the sphere (on the right) after running for 1 hour. The top row is for zero gravity (eccentricity of 0), the middle row for the Moon (eccentricity of 0.25 resulting in partial gravity of 0.17) and the bottom row for Mars (eccentricity of 0.53 resulting in a partial gravity of 0.38). The scale bars represent the normalized density where 1 is uniform density. One wants the pictures on the left to be as uniform as possible. For the pictures on the right it depends on the desired partial gravity factor. Mean is the mean error between the desired and the actual partial gravity factor. Sigma is the standard deviation of the normalized density of the distribution relative to a uniform distribution.*

**References**

1 van Loon, J. J. W. A. (2007). Some history and use of the Random Positioning Machine, RPM, in gravity related research. Adv Space Res 39, 1161-5.

2 Borst, A. G., van Loon, J. J. W. A. (2009). Technology and developments for the random positioning machine, RPM. *Microgravity Sci. Technol* **21**, 6.

3 Hasenstein, K. H., and van Loon, J. J. W. A. (2015). Clinostats and other rotating systems — Design, function, and limitations. in “Generation and Applications of Extra-Terrestrial Environments on Earth”, D.A. Beysens & J.J.W. A. van Loon (eds.). *River Publishers, Denmark.*

4 Wuest S.L., Richard S., Walther I., R Furrer R., Anderegg R., Sekler J., Egli M. (2014) A novel microgravity simulator applicable for three dimensional cell culturing. Microgravity Science and Technology, **26(2)**, 77 – 88.

5 Kim Y.J., Jeong A.J., Kim M., Lee C., Ye S.K., Kim S. (2017) Time‑averaged simulated microgravity (taSMG) inhibits proliferation of lymphoma cells, L‑540 and HDLM‑2, using a 3D clinostat. Biomed Eng Online. **16:48**, 1-12.

6 Perbal G., Jeune B., Lefranc A., Carnero-Diaz E., Driss-Ecole D. (2002) The dose–response curve of the gravitropic reaction: a re-analysis. Physiol Plant. **114(3)**, 336-342.

7 Lebert M., Porst M., Richter P, Hader D.P. (1999) Physical characterization of gravitaxis in Euglena gracilis. J Plant Physiol. **155(3)**, 338-343.

8 Jørgensen E. (1975) The central limit problem for geodesic random walks. Zeitschrift für Warscheinlichkeitstheorie und Verwandte Gebiete. **32(1)**, 1 – 64.
